# The Sports Cardiology Team: Personalizing Athlete Care Through a Comprehensive, Multidisciplinary Approach

**DOI:** 10.1016/j.mayocpiqo.2022.08.006

**Published:** 2022-10-12

**Authors:** Juliette C. van Hattum, Sjoerd M. Verwijs, P. Jeff Senden, Jessica L. Spies, S. Matthijs Boekholdt, Maarten Groenink, Nicole M. Panhuyzen-Goedkoop, Albert R. Willems, Ingmar Knobbe, Nicolaas A. Blom, Cornelis A.C.M. Wijne, Gustaaf Reurink, Saskia N. van der Crabben, Nick R. Bijsterveld, Evert A.L.M. Verhagen, Yigal M. Pinto, Arthur A.M. Wilde, Harald T. Jørstad

**Affiliations:** a Department of Cardiology, University of Amsterdam, Amsterdam UMC, Amsterdam, the Netherlands; b Department of Paediatric Cardiology, and University of Amsterdam, Amsterdam UMC, Amsterdam, the Netherlands; c Department of Clinical Genetics, University of Amsterdam, Amsterdam UMC, Amsterdam, the Netherlands; d Department of Cardiology, Meander Medical Center, Amersfoort, the Netherlands; e Department of Cardiology, Sports Medical Centre Papendal, Arnhem, the Netherlands; f Department of Cardiology and Hospital Onze Lieve Vrouwe Gasthuis, Amsterdam, the Netherlands; g Department of Sports Medicine, Hospital Onze Lieve Vrouwe Gasthuis, Amsterdam, the Netherlands; h Department of Cardiology, Flevoziekenhuis, Almere, the Netherlands; i Department of Epidemiology of Sports, Exercise & Health, Amsterdam UMC, Amsterdam, the Netherland

**Keywords:** CVD, cardiovascular disease, ICD, implantable cardioverter-defibrillator, MDT, multidisciplinary team, SEM, sports and exercise medicine, Amsterdam UMC, Amsterdam University Medical Centers

## Abstract

**Objective:**

To systematically investigate and document the infrastructure, practices, recommendations, and clinical consequences of a structured, organized sports cardiology multidisciplinary team (MDT) for athletes and patients who wish to engage in sports and exercise.

**Patients and Methods:**

We established bimonthly sports cardiology MDT meetings, with a permanent panel of experts in sports cardiology, genetics, pediatric cardiology, cardiovascular imaging, electrophysiology, and sports and exercise medicine. Cases were referred nationally or internationally by cardiologists/sports physicians. We retrospectively analyzed all MDT cases (April 10, 2019 through May 13, 2020) and collected clinical follow-up data up to 1 year after the initial review.

**Results:**

A total of 115 athletes underwent MDT review; of them, 11% were women, 65% were recreational athletes, and 54% were performing “mixed” type of sports; the mean age was 32±16 years. An MDT review led to a diagnosis revision of “suspected cardiac pathology” to “no cardiac pathology” in 38% of the athletes and increased the number of definitive diagnoses (from 77 to 109; *P*=.03). We observed fewer “total sports restrictions” (from 6 to 0; *P*=.04) and more tailored sports advice concerning “no peak load/specific maximum load” (from 10 to 26; *P*=.02). At the 14±6-month follow-up, 112 (97%) athletes reported no cardiovascular events, 111 (97%) athletes reported no (new) cardiac symptoms, 113 (98%) athletes reported adherence to the MDT sports advice, and no diagnoses were revised.

**Conclusion:**

Our experiences with a comprehensive sports cardiology MDT demonstrate that this approach leads to higher percentages of definitive diagnoses and fewer cardiac pathology diagnoses, more tailored sports advice with excellent rates of adherence, and fewer total sports restrictions. Our findings highlight the added value of sports cardiology MDTs for patient and athlete care.

Multidisciplinary teams (MDTs) are an integral part of general medicine, cardiology, and sports and exercise medicine (SEM). In cardiology, MDTs are mostly organized according to the type of pathology, such as coronary artery disease, valve pathology, arrhythmias, and congenital heart disease. In SEM, MDTs most commonly address musculoskeletal problems. In general medicine, MDTs have been shown to enhance decision making and improve the quality of care and patient management.^1^^,^^2^ In clinical care for competitive athletes and highly active individuals, the American College of Cardiology recommends involving sports cardiologists,^3^ and the American Medical Society for Sports Medicine has highlighted the need for multidisciplinary networks within sports cardiology and SEM.^4^

Although the infrastructure, processes, and effects of most cardiology MDTs have been published and are commonly recommended or mandated by international guidelines, to date, there are no published studies on sports cardiology MDTs and no specific recommendations to guide the optimal development of such MDTs. Sports cardiology occupies the intersection between cardiology and SEM and mandates both broad and highly specific cardiology expertise in addition to sports-specific expertise. Owing to the generally low level of evidence in the group of athlete patients, expert opinion and consensus occupy a central position in both the diagnostic process and formulation of sports advice. Furthermore, restrictive sports advice can have considerable impact, especially in elite and highly motivated recreational athletes. Therefore, we established a formally organized sports cardiology MDT at the Amsterdam University Medical Centers (UMC) in April 2019, aiming to facilitate the diagnostic process, enhance the process of formulating optimal sports advice, and maximize safety in sports.

With this study, we systematically investigated and documented the practices, recommendations, and clinical consequences of our formally organized sports cardiology MDT to serve as a proof of concept for the care of elite and recreational athletes and individual patients who wish to engage in sports and exercise.

## Methods

### Study Design

We retrospectively analyzed all the reviewed athlete patients of our (online) bimonthly sports cardiology MDT meetings from April 10, 2019 through May 13, 2020. All athletes provided consent to their primary caregiver to be discussed by the MDT. A waiver was granted by the Institutional Review Board of the Amsterdam UMC, exempting our study from the need to obtain written informed consent. Owing to the retrospective design of our descriptive study, patients were not exposed to any health risks or additional burdens. The primary objective of this study was to investigate the 2-year experiences of the documented practices, recommendations, and clinical consequences of a formal sports cardiology MDT.

### Multidisciplinary Team Design and Infrastructure

**Figure 1:**
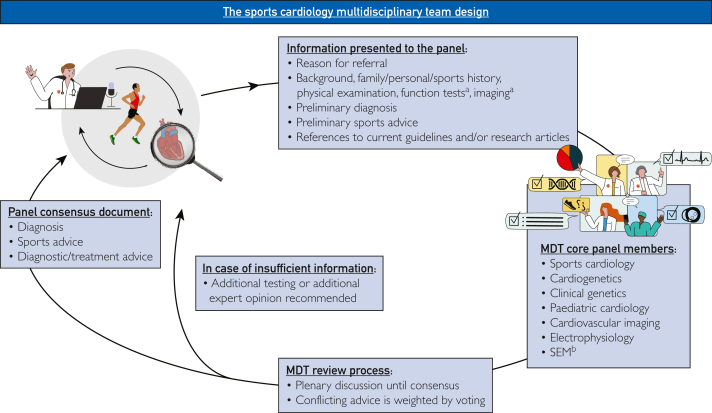
Process diagram illustrating the sports cardiology MDT design. ^a^Including complete clinical examination and imaging studies. ^b^Sports and exercise medicine professionals with extensive experience in elite and recreational sports, next to specific expertise in football, endurance running, cycling, swimming, altitude sports, and diving. MDT, multidisciplinary team; SEM, sports and exercise medicine.

The MDT meeting is organized by the Amsterdam UMC, the Netherlands, and is a hybrid meeting, with physical and digital attendance. Cases of athletes and athlete patients are contributed by cardiologists or SEM professionals or referred nationally or internationally by other affiliated medical professionals. Before MDT review, all cases were evaluated by a health care professional (eg, for preparticipation screening, symptoms during/after exercise, or known cardiovascular disease (CVD) with potential interaction with sports and exercise), and, when deemed necessary by the individual health care professionals, were referred to the MDT for expert opinion or consensus advice. Typically, 1 MDT meeting consists of an extensive case review and discussion of up to 4 cases, with regular feedback or updates on previously discussed cases. Cases are prepared as digital presentations according to a structured format (Figure 1). Information presented to the panel comprises medical and sports history, family history, physical examination findings, additional diagnostic test results (eg, cardiac imaging, function testing, laboratory results, etc), clinical data collected during follow-up, the preliminary diagnosis, the preliminary sports advice, the reason for referral, and references to current guidelines or research papers. No information is blinded to the MDT. The MDT consists of a permanent core panel of experts from different fields of sports cardiology, cardiogenetics, clinical genetics, pediatric cardiology, cardiovascular imaging, electrophysiology, and SEM (involved in preparticipation screenings and elite/competitive care) and is chaired by a dedicated sports cardiologist. At least 1 expert in each of the core disciplines is present at every meeting. Generally, the number of experts and guests per meeting varies between 15 and 30, and the team consists of at least 2 experts in the field of SEM with extensive experience in elite and recreational sports, next to specific expertise in football, endurance running, cycling, swimming, altitude sports, and diving. For specific cases, other experts in relevant fields are invited (eg, gender-specific expertise, congenital cardiology, interventional cardiology, pulmonology, cardiothoracic surgery, highly specific sports expertise, etc). The MDT also includes a health care professional with a personal background in elite sports, whose specific role is to highlight the athletes’ perspectives. Each case is registered in the Amsterdam UMC electronic patient record. Multidisciplinary team considerations, consensus, conclusions, and recommendations are documented in the electronic patient record and communicated to the referring health care professionals. Consensus is reached through a plenary discussion; in cases where there is inadequate information to reach a conclusion or consensus, additional testing or the input of an additional expert is recommended. If a consensus cannot be reached, the conflicting advice is weighted by voting (ie, advice A recommended by 80% of the MDT, advice B recommended by 20% of the MDT, etc.) and communicated back to referring health care professionals. Referring health care professionals are specifically asked to discuss the MDT advice—in particular, the MDT sports advice—with individual athletes, taking the context of personal ambitions and preferences into account. Finally, referring health care professionals are asked to monitor cases regularly according to relevant guidelines^5^ and provide feedback to the MDT in case of clinical changes, such as new complaints or cardiac events.

### Study Population

We included all MDT-reviewed athletes or athlete patients who wished to engage in sports from April 2019 up to January 2021. Athletes were defined as individuals, either amateur or elite, who engage (or plan to engage) in regular exercise training or participate in official sports competitions.^5^ Elite athletes (ie, national team, Olympians, Paralympians, and professional athletes) were defined as athletes who participate at the highest level of national or international competitions (>10 h/wk). Recreational athletes were defined as individuals who engage (or plan to engage) in sports for pleasure/leisure-time activity (>3 h/wk) with or without a competitive component.^5^

### Data Collection

Data were collected from the electronic patient record of each MDT-reviewed case. First, we extracted demographic data on age, sex, type of sport (classified as skill-, power-, mixed-, and endurance-type according to the European Society of Cardiology guideline),^5^ levels of sports (recreational/elite), weekly hours of exercise/sports, and reason for MDT review. Second, we collected data on the diagnosis, medication, and sports advice at MDT application and those after the MDT review. Third, after the MDT review, we extracted individual MDT recommendations for cardiovascular management, such as additional testing and interventions. We extracted the final revisions of the diagnosis and sports advice after such additions, as appropriate. Finally, follow-up data were collected, consisting of time since the initial MDT review, self-reported adherence to the MDT sports advice, and relevant (new) symptoms, such as anxiety, dyspnea, chest discomfort, syncope/near-syncope, reduced exercise performance, and palpitations. We also collected data on cardiovascular events and hospitalizations (defined as ≥24 hours of hospital stay). For individuals reviewed by the MDT on 2 or more occasions, we extracted data on diagnosis and sports advice from the final MDT meeting.

### Statistical Methods

Data are expressed as mean ± standard deviation or frequencies and percentages, as appropriate. The Shapiro–Wilk test method was used to assess whether variables were normally distributed. The paired or unpaired sample *t*-test, chi-square test, and Fisher exact test were used to investigate differences between recreational and elite athletes, as appropriate. The Wilcoxon rank sum test was used to investigate the difference between diagnoses and sports advice before and after the MDT review. Transition plots were used to illustrate how diagnoses and sports advice changed before and after the MDT review. A *P* value of less than .05 indicated statistical significance. All analyses were performed using R statistical software (version 1.3.1993).

## Results

We included 115 athletes that underwent an MDT review between April 2019 and May 2020. Follow-up data were collected up to 1 year after the initial review and were available in all cases, and no athletes were lost to follow-up during or after the MDT review process. The mean age was 32±16 years; of the 115 athletes, 11% (n=13) were women, and 65% (n=75) were recreational athletes, and 35% (n=40) were elite athletes (Table 1). The mean age of the recreational athletes was higher than that of the elite athletes (37 vs 23 years; *P*=.01). The majority of cases (54%) engaged in a mixed type of sports. Recreational athletes trained significantly lesser than elite athletes (6.5 vs 13.7 h/wk; *P*=.01). The main reasons for referral (as reported by referring health care professionals) to the MDT were as follows: (1) 48% (n=55) personalized sports advice (eg, should this athlete be restricted for all types of sports or only for aquatic sports or a specific peak load/competition or should we advise no restrictions?), (2) 27% (n=31) expert opinion for definitive diagnosis, and (3) 25% (n=29) expert advice for abnormal diagnostic findings (ie, electrocardiography, cardiac magnetic resonance imaging, cardiopulmonary exercise testing, transthoracic echocardiography, exercise transthoracic echocardiography, or myocardial perfusion imaging abnormalities). Of the 48% of athletes referred for personalized sports advice, 16% were asymptomatic athletes with an abnormal preparticipation screening, 37% were symptomatic athletes with (a suspicion of) CVD, and 47% were asymptomatic athlete patients with CVD.

**Table 1:** General Characteristics of MDT Cases

	Total (N=115)	Recreational (N=75)	Elite (N=40)	*P* Value
Age (y), mean ± SD	32±16	37±17	23±6	.01
Women, n (%)	13 (11)	7 (9)	6 (15)	.87
Sport/exercise (h/wk), n (%)				
0-5	36 (31)	36 (48)	0 (0)	<.001
6-10	31 (27)	28 (37)	3 (8)	<.001
>10	48 (42)	11 (15)	37 (92)	<.001
Sports history ESC classification, n (%)				
Endurance	40 (35)	32 (43)	8 (20)	.02
Mixed^a^	64 (54)	33 (43)	31 (78)	<.001
Power	8 (7)	7 (10)	1 (2)	.26
Skill	1 (1)	1 (1)	0 (0)	.92
No sports^b^	2 (3)	2 (3)	0 (0)	.88
Reason for MDT review, n (%)				
Abnormal diagnostic test	29 (25)	13 (17)	16 (40)	.01
Expert opinion for definitive diagnosis	31 (27)	19 (25)	12 (30)	.75
Sports advice	55 (48)	43 (58)	12 (30)	<.001
Asymptomatic abnormal preparticipation screening	9 (16)	9 (20)	0 (0)	.18
Symptomatic with suspicion of CVD	20 (37)	17 (40)	3 (25)	.50
Asymptomatic with known CVD	26 (47)	17 (40)	9 (75)	.06

CVD, cardiovascular disease; ESC, European Society of Cardiology; MDT, multidisciplinary team.

a Including occupational sports (ie, special weapons and tactics).

b Wished sports advice before initiation of sports.

### Multidisciplinary Team Review

**Figure 2:**
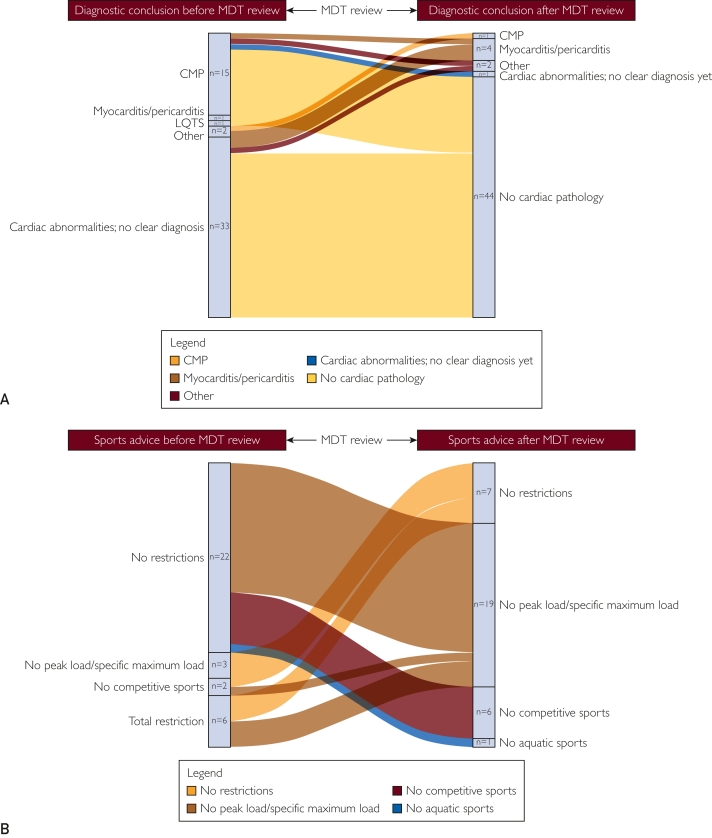
A, A transition plot illustrating revised diagnostic conclusion before (left bar) and after (right bar) MDT review in 45% (52 of 115) of athletes (an unchanged diagnostic conclusion not shown). B, A transition plot illustrating revised sports advice before (left bar) and after (right bar) MDT review in 29% (33 of 115) of athletes (unchanged sports advice not shown). CMP, cardiomyopathy; LQTS, long QT syndrome; MDT, multidisciplinary team.

The main findings before and after the MDT review are summarized in Table 2. Diagnosis revision took place in 52 (45%) athletes (Figure 2A). Overall, the main diagnoses revised were “suspicion of (nonspecified) cardiomyopathy” to “no cardiac pathology” in 12 (21%) athletes and “cardiac abnormalities with no clear diagnosis” to “no cardiac pathology” in 30 (26%) athletes (Figure 2A). After the MDT review, we observed an increase in the number of confirmed diagnoses (before vs after the MDT review, 77 vs 109; *P*=.03). The total number of cases with a diagnosed cardiomyopathy was revised from 42 (37%) before the MDT review to 28 (24%) after the MDT review (*P*=.08). Furthermore, the MDT recommended additional imaging studies in 13 (11%) athletes and genetic testing in 13 (11%). Pharmacological therapy was already recommended by referring health care professionals in 6 (5%) athletes before the MDT review, and it was recommended in an additional 8 (7%) athletes after the MDT review. Finally, the MDT advised internal loop recorder implantation in 3 athletes (1 recreational athlete and 2 elite athletes) with syncope during sports; among these 3 athletes, 1 had underlying (active) perimyocarditis with scarring, 1 had unexplained syncope, and 1 had transient loss of consciousness. An implantable cardioverter-defibrillator (ICD) was implanted in an additional 3 athletes (2 recreational athletes and 1 elite athlete) with structural heart disease with arrhythmogenic substrate and a high risk of ventricular arrhythmias (hypertrophic cardiomyopathy, n=1; and perimyocarditis, n=2).

**Table 2:** Characteristics Before and After the MDT Review

	Total (N=115)			Recreational (N=75)			Elite (N=40)		
	Before MDT	After MDT	*P* Value	Before MDT	After MDT	*P* Value	Before MDT	After MDT	*P* Value
Diagnosis, n (%)									
Cardiac abnormalities; no clear diagnosis	38 (33)	6 (5)	.03	24 (32)	6 (8)	.01	14 (35)	0 (0)	.04
CMP	42 (37)	28 (24)	.08	30 (40)	20 (27)	.22	12 (30)	8 (20)	.42
Congenital disease^a^	9 (8)	9 (9)	.92	5 (7)	5 (7)	.94	4 (10)	4 (10)	.94
LQTS	6 (6)	5 (4)	.97	4 (5)	3 (4)	1.00	2 (5)	2 (5)	1.00
Myocarditis/Pericarditis	9 (7)	12 (11)	.90	5 (7)	8 (11)	.91	4 (10)	4 (10)	1.00
No cardiac pathology/physiological adaptation	0 (0)	44 (38)	.001	0 (0)	25 (33)	.001	0 (0)	19 (48)	.001
Other^b^	11 (10)	11 (10)	.94	7 (9)	8 (11)	.96	4 (10)	3 (8)	.90
Medication, n (%)	25 (22)	31 (27)	.43	20 (27)	24 (32)	.64	5 (13)	7 (18)	.86
Sports advice, n (%)									
Total restriction	6 (5)	0 (0)	.04	4 (5)	0 (0)	.06	2 (5)	0 (0)	.08
No competitive sports	7 (6)	11 (10)	.57	7 (10)	9 (12)	.98	0 (0)	2 (5)	.85
No peak load/specific maximum load	10 (9)	26 (23)	.02	10 (13)	25 (33)	.03	0 (0)	1 (2.5)	.90
No aquatic sports	0 (0)	1 (1)	.98	0 (0)	1 (1)	.99	0 (0)	0 (0)	.93
No restrictions	92 (80)	77 (67)	.01	54 (72)	40 (53)	.01	38 (95)	37 (92)	.85

CMP, cardiomyopathy; LQTS, long QT syndrome; MDT, multidisciplinary team.

a Atrial septal defect (n=2), myocardial bridging (n=1), bicuspid aortic valve (n=3), right coronary artery split (n=1), tricuspid valve dysplasia (n=1), and total anomalous pulmonary venous connection (n=1).

b Sarcoidosis (n=1), type 1 Brugada syndrome (n=1), familial thoracic aortic aneurysm and dissection (n=1), familial hypercholesterolemia (n=1), premature atherosclerosis (n=1), left ventricle hypertrophy (n=1), chronic total occlusion obtuse marginal artery (n=1), paroxysmal atrial fibrillation (n=3), and hypertension (n=1).

The MDT review significantly impacted the number of athletes with negative sports advice; we observed a decrease in “total sports restriction” after the MDT review (before vs after, 6 vs 0; *P*=.04) and more specific/tailored sports advice concerning “no peak load/specific maximum load” (before vs after the MDT review, 10 vs 26; *P*=.02) (Figure 2B). Overall, sports advice was revised to more tailored advice (ie, no competitive sports, no peak load/specific maximum load, and no aquatic sports) in 33 (29%) athletes. The advice “no restrictions” was revised to “no peak load/specific maximum load” in 15 (45%) of 33 athletes, and the advice “no restrictions” was revised to “no competitive sports” in 6 (18%) of 33 athletes.

### Recreational vs Elite Athletes

In total, 75 (65%) recreational athletes and 40 (35%) elite athletes were included in our study. The diagnosis was revised after the MDT review in nearly 50% of the respective groups (50% vs 43%; *P*=.87). In addition, in both of the groups, the number of “cardiac abnormalities, no clear diagnosis” significantly decreased after the MDT review; in recreational athletes, it decreased from 32% before the MDT review to 8% after the MDT review (*P*=.05), and in elite athletes, it decreased from 35% before the MDT review to 0% after the MDT review (*P*=.05). Revision of the diagnosis concerning “cardiac abnormalities with no clear diagnosis” to “no cardiac pathology” occurred twice as often in recreational athletes (65%) as in elite athletes (30%) (*P*=.01). A definitive diagnosis of “no cardiac pathology” was given less frequently in recreational athletes (33%) than in elite athletes (48%) (*P*=.01).

After the MDT review, no recreational athletes received negative sports advice amounting to a “total sports restriction” (before vs after the MDT review, n=4 vs n=0; *P*=.06), with an increase in more specific sports advice concerning “no peak load/specific maximum load” (before vs after the MDT review, n=10 vs n=25; *P*=.03). Furthermore, additional imaging was recommended in 11% (n=8) of the recreational athletes and 10% (n=4) of the elite athletes, genetic testing was recommended in 11% (n=8) of the recreational athletes, and (additional) pharmacological therapy was recommended in 5% of both recreational and elite athletes.

### Follow-up

At a mean follow-up of 14±6 months after the initial MDT review, clinical events/hospitalizations were reported in 3 (3%) athletes and (new) cardiac symptoms were reported in 4 (3%) athletes, of which 2 athletes were symptomatic at the time of initial referral (Table 3; and details can be found in Supplemental Table 1, available online at http://www.mcpiqojournal.org). The referring health care professionals reported adherence to MDT sports advice to be adequate in 113 (98%) athletes. No diagnoses were revised during follow-up. In total, 2 (2%) athletes required hospitalization. One recreational athlete with a diagnosis of exercise-induced arrhythmogenic right ventricular cardiomyopathy after the MDT review chose to ignore the MDT recommendations and sports advice. He subsequently experienced a syncopal event during a sport activity, was hospitalized, and underwent ICD implantation. After the MDT review, 1 elite athlete was hospitalized for arrhythmia monitoring, previously diagnosed as supraventricular tachycardia, and subsequently underwent radiofrequency ablation of an uncommon type of atrioventricular nodal reentrant tachycardia. Finally, 1 elite athlete who underwent ICD implantation after the MDT review had a single appropriate ICD discharge during a sport activity after returning to play.

**Table 3:** Characteristics of Follow-Up

	Total (N=115)	Recreational (N=75)	Elite (N=40)
Follow-up (mo), mean ± SD	14±6	13±6	16±7
Adherence to sports advice, n (%)	113(98)	74 (99)	39 (98)
New symptoms, n (%)			
Anxiety	2 (2)	1 (1)	1 (2)
Palpitations	1 (1)	1 (1)	0 (0)
Nonanginal chest pain	1 (1)	1 (1)	0 (0)
Hospitalization/Clinical events, n (%)			
Syncope, followed by ICD implantation	1 (1)	1 (1)	0 (0)
Uncommon AVNRT RF ablation	1 (1)	0 (0)	1 (2)
Appropriate ICD shock	1 (1)	0 (0)	1 (2)

AVNRT, atrioventricular nodal reentrant tachycardia; ICD, implantable cardioverter-defibrillator; RF, radiofrequency.

## Discussion

This study demonstrates that a structured sports cardiology MDT leads to a significantly higher percentage of definitive diagnoses, with a major impact on case reclassification and with suspected cardiac pathology frequently being reclassified as physiological adaptation. Moreover, our findings highlight that a (multidisciplinary) team-based approach leads to a lower number of total sports restrictions and more tailored sports advice. Finally, we observed a high level (98%) of adherence to the MDT sports advice and numerous instances of more tailored cardiovascular management in addition to a low percentage of (new) symptoms, cardiovascular events, or hospitalizations after more than a year of follow-up. To our knowledge, our study is the first to systematically investigate and document the practices, recommendations, and clinical consequences of a sports cardiology MDT. Moreover, our study indicates that a sports cardiology MDT approach safely contributes, in a clinically meaningful manner, to more tailored and personalized care for athletes and patients who wish to engage in sports.

The field of sports cardiology has demonstrated rapid progression in the 2 last decades, with numerous consensus documents,^6^, ^7^, ^8^, ^9^ a recent European guideline,^5^ and several landmark studies becoming available to assist clinicians in differentiating between pathology and physiological cardiovascular adaptation to sports.^10^^,^^11^ Nevertheless, expert opinion remains indispensable for individual cases, as illustrated by the fact that the current European Society of Cardiology sports cardiology guidelines include 12 “level of evidence A” recommendations, against 126 “level of evidence C” recommendations (ie, expert consensus). Our study demonstrates that a sports cardiology MDT facilitates the process of providing expert consensus recommendations for individual cases. Furthermore, we demonstrated high reclassification rates (45%) of findings, initially interpreted as cardiovascular abnormalities or even clear pathology, such as cardiomyopathies to cardiac adaptation to sports with no evidence of cardiovascular pathology. Therefore, a formally structured sports cardiology MDT is complementary to guideline-based care and is of added value to clinical care for athletes and patients, especially for the differentiation between pathology and physiology.

After the MDT review, sports advice was revised in 29% of the athletes. In patients without a diagnosis of cardiac pathology after the MDT review, this advice was nonrestrictive. In contrast, individuals in whom cardiac pathology was diagnosed were given more tailored and specific advice. This lower rate of total sports restrictions and a higher rate of personalized advice may reflect the broad spectrum of expertise available in the panel, with both SEM and cardiological input, with additional expertise for specific cases. Moreover, individually tailored sports advice may have contributed to the high levels of self-reported adherence to the MDT sports advice (98%) at the 1-year follow-up.

Ideally, long-term follow-up of the MDT review and clinical consequences should be performed. Our mean follow-up period was 14 months; yet, in this follow-up period, next to excellent adherence to the MDT sports advice, no diagnoses were revised on the basis of new clinical findings, and the number of new complaints or cardiac events was modest. Only 2 athletes reported not adhering to their sports advice; of these 2, 1 athlete with an exercise-induced arrhythmogenic right ventricular cardiomyopathy had exercise-related syncope, followed by ICD implantation. Furthermore, our study included a considerable number of elite athletes who engaged in intensities and exercise volumes far above those of the recreational athletes, and within this group, only 1 cardiac adverse event occurred during the follow-up period. Considering the major impact of restrictive sports advice on professional and elite athletes’ careers, the addition of even a limited number of unrestricted sport years may be of value, both personally and professionally.

### Strengths and Limitations

This study has several strengths. First, we included a considerable number of consecutive cases of recreational and elite athletes, with complete follow-up data in all cases. Second, each case was thoroughly documented in the electronic patient record during the MDT review and linked back to the referring health care professional, and we continued monitoring over time, with MDT updates in case of new symptoms or events. Finally, to our knowledge, this is the first study investigating the infrastructure, management recommendations, and clinical consequences of a sports cardiology MDT, which contributes to clarifying the clinical knowledge gaps in sports cardiology.

Some aspects of our study warrant consideration. First, this study included only patients referred specifically for MDT review; hence, per definition, a referral bias cannot be excluded. This may potentially have been one factor influencing the lack of gender diversity. Although this might somewhat limit generalizability, this does not invalidate the process of the MDT itself. Second, for a number of the diagnoses revised by the MDT, no “golden” diagnostic standard exists. Our MDT de facto fulfilled the role of an expert panel of diagnostic research. As such, validation of our findings by an additional diagnostic panel would have little added value, especially considering that, commonly, such panels consist of only 2 or 3 members.^1^ To date, most other cardiovascular MDTs have not included such validation. Our MDT panel had access to all diagnostic test results and clinical information of individual cases. We documented how consensus was reached and how conflicting advice was presented, and a large number of experts from different types of fields were represented. However, we did not assess the reproducibility of the decision-making process of our MDT. Yet, the fact that no MDT diagnoses were revised during clinical follow-up provides some insight into the validity of the MDT decisions.^1^ Third, symptoms and events not requiring contact with a health care professional were self-reported, which potentially could have led to an underestimation of mild or transient new symptoms and potentially the accuracy of reclassified diagnosis. However, each case was intensively monitored by referring health care professionals, who provided feedback to the MDT about clinical changes, reducing the likelihood of missing important clinical changes. Finally, a longer follow-up period is needed to assess the long-term consequences of the MDT’s diagnostic revisions and sports advice.

## Conclusion

Our experiences with a comprehensive, multidisciplinary sports cardiology MDT demonstrate that such an approach leads to more personalized treatment and sports advice for both recreational and elite athletes. The team-based approach leads to a higher percentage of definitive diagnoses, more frequent revisions to “no cardiac pathology,” fewer total sports restrictions, and high adherence to MDT sports advice. Our findings serve as a proof of concept of the added value of the sports cardiology team in care for elite, professional, and recreational athletes and patients who wish to engage in sports and exercise.

## Potential Competing Interests

The authors report no competing interests.
