# Supplementary material for: The Sports Cardiology Team: Personalizing Athlete Care Through a Comprehensive, Multidisciplinary Approach
Source: Mayo Clin Proc Innov Qual Outcomes. 2022 Oct 12;6(6):525–35. doi: 10.1016/j.mayocpiqo.2022.08.006 (PMC9576626; doi:10.1016/j.mayocpiqo.2022.08.006)
Supplement: Supplemental material [file mmc1.docx]

**SUPPLEMENTAL MATERIAL**

Table 1. Individual information of cases who experienced symptoms/events or hospitalisation* during follow-up

| **Age** | **Gender** | **Level of sports** | **Initial reason for MDT referral** | **Diagnosis after MDT** | **Follow-up** | | | |
| --- | --- | --- | --- | --- | --- | --- | --- | --- |
|  |  |  |  |  | **Adhered to sports advise** | **Symptoms** | **Events** | **Hospitalisation^a^** |
| 30 | Man | Elite | Personalised sports advice in an asymptomatic athlete-patient with CVD | Premature atherosclerosis | Yes | Anxiety | - | - |
| 37 | Man | Recreational | Personalised sports advice in an asymptomatic athlete with an abnormal pre-participation screening | Dilated Cardiomyopathy | Yes | Anxiety | - | - |
| 54 | Man | Recreational | Personalised sports advice in an asymptomatic athlete with abnormal pre-participation screening | Peri-/myocarditis (recovered) | Yes | Non-anginal chest pain | - | - |
| 34 | Man | Recreational | Personalised sports advice in an asymptomatic athlete-patient with CVD | Paroxysmal atrial fibrillation | Yes | Palpitations during sports | - | - |
| 31 | Man | Elite | Expert opinion regarding symptoms and diagnosis | Myocarditis (recovered) | Yes | - | Appropriate ICD discharge | - |
| 67 | Man | Recreational | Personalised sports advice in a symptomatic athlete-patient with (suspicion of) CVD | ARVC | No | Syncope | - | ICD implantation; based on cardiac syncope during sports |
| 20 | Man | Elite | Consultation for discussing an asymptomatic athlete with abnormal heart-rate-monitor results during exercise | Uncommon type AVNRT | Yes | Palpitations | - | RF-ablation (slow pathway modification) |

*^a^Hospitalisation was defined as ≥24 hours hospital stay.
ICD; implantable cardioverter defibrillator, ARVC; arrhythmogenic right ventricle cardiomyopathy, AVNRT; Atrioventricular nodal re-entry tachycardia, RF-ablation; radiofrequency ablation, CVD; Cardiovascular disease*
